# Comprehensive analysis reveals *COPB2* and *RYK* associated with tumor stages of larynx squamous cell carcinoma

**DOI:** 10.1186/s12885-022-09766-z

**Published:** 2022-06-17

**Authors:** Guojin Zhou, Shoude Zhang, Mao Jin, Sunhong Hu

**Affiliations:** Department of Otolaryngology Head and Neck Surgery, Sir Run Run Shaw Hospital, College of Medicine, Zhejiang University, No.3 Qingchun East Road, Hangzhou, 310016 Zhejiang China

**Keywords:** LSCC, WGCNA, Immune infiltration, Tumor stages, GSEA

## Abstract

**Background:**

Laryngeal squamous cell carcinoma (LSCC) is one of the highly aggressive malignancy types of head and neck squamous cell carcinomas; genes involved in the development of LSCC still need exploration.

**Methods:**

We downloaded expression profiles of 96 (85 in advanced stage and 11 in early stage) LSCC patients from TCGA-HNSC. Function enrichment and protein-protein interactions of genes in significant modules were conducted. Univariate and multivariate Cox regression analyses were performed to explore potential prognostic biomarkers for LSCC. The expression levels of genes at different stages were compared and visualized via boxplots. Immune infiltration was examined by the CIBERSORTx web-based tool and depicted with ggplot2. Gene set enrichment analysis (GSEA) was utilized to analyze functional enrichment terms and pathways. Immunohistochemical staining (IHC) was used to verify the expression of genes in the LSCC samples.

**Results:**

We identified 25 modules, including 3 modules significantly related to tumor stages of LSCC via weighted gene co-expression network analysis (WGCNA). *UIMC1*, *NPM1*, and *DCTN4* in the module ‘cyan’, *TARS* in the module ‘darkorange’, and *COPB2* and *RYK* in the module ‘lightyellow’ showed statistically significant relation to overall survival. The expression of *COPB2*, *DCTN4*, *RYK*, *TARS*, and *UIMC1* indicated association with the change of fraction of immune cells in LSCC patients; two genes, *COPB2* and *RYK,* indicated different expression in various tumor stages of LSCC. Finally, *COPB2* and *RYK* showed high-expression in tumor tissues of advanced LSCC patients.

**Conclusions:**

Our study provided a potential perceptive in analyzing progression of LSCC cells and exploring prognostic genes.

## Introduction

Laryngeal squamous cell carcinoma (LSCC) is one of the most frequent types of head and neck squamous cell carcinomas (HNSCC) and is the fifth cause leading to tumor-related death worldwide [1]. Patients with LSCC still have a poor prognosis and survival due to its recurrence and metastasis, even after improvement with surgical intervention, chemotherapy, and radiotherapy [2, 3]. Most patients were diagnosed at stage III or stage IV, making the poor prognosis even worse [1].

Several publications reported molecular signatures associated with different stages of LSCC patients. *BCL2L12* was reported to be a favorable prognostic tissue biomarker in patients with primary advanced-stage LSCC [4]. The long non-coding RNA *CDKN2B-AS1* facilitates LSCC by regulating miR-497/CDK6 pathway and is associated with advanced clinical stage [5]. Patients with lymph node metastasis/advanced clinical stages and high Wnt3a expression had worse overall survival rates than patients with other features [6]. High FTH1P3 expression, positively correlated with advanced clinical stage, predicts a poor prognosis, and promotes aggressive phenotypes of LSCC [7]. Downregulation of P38 phosphorylation correlates with low-grade differentiation, proliferation, and advanced stage of LSCC [8]. The prognostic value of genes and underlying molecular mechanisms related to various stages of LSCC patients, however, is limited.

Weighted gene co-expression network analysis (WGCNA) was frequently used to construct networks, detect module, and relate modules and genes to external information [9]. It has been widely performed to describe the association between genes and sequencing samples in various cancers including LSCC. WGCNA was utilized to analyze the co-expression network and identify genes associated with cancer stem cell characteristics in LSCC [10]. *TPX2*, *MCM2*, *UHRF1*, *CDK2*, and *PRC1* were found to hold a possible association with LSCC using WGCNA [11]. However, more work needs to be done in exploring the prognostic biomarkers, molecular signatures and mechanisms, such as gene modules or networks, in different tumor stages of LSCC patients.

In this study, we aimed to find significant genes networks related to different stages of LSCC and explored novel genes with prognostic significance. Tumor stage related modules was detected using WGCNA; the functional enrichment analysis and protein-protein interaction (PPI) were performed to analyze function and interactions of genes associated with LSCC stages. Univariate Cox regression and multivariate Cox regression analysis were conducted to explore prognostic signature of LSCC; the immune response and gene set enrichment analyses were conducted to characterize the potential mechanism of genes related to various tumor stage of LSCC.

## Materials and methods

### Data collection and processing of TCGA

Gene expression profiles were downloaded from the TCGA database; since LSCC patients were involved in HNSC (head-neck squamous cell carcinoma) project in TCGA (https://portal.gdc.cancer.gov/projects/TCGA-HNSC), we downloaded expression profiles using ‘TCGA-HNSC’ as query using GDC data transfer tool (gdc-client); duplicated samples were discarded; HTSeq (a Python package calculating the number of mapped reads of genes) counts of samples were downloaded from TCGA-HNSC; LSCC-related expression matrix in tumor tissues (96 samples) was collected for this research. HTSeq counts of RNA-seq data were transformed into the trimmed mean of M-values (TMM) with the *edgeR* package [12] and transformed with ‘voom’ method in *limma* package [13] to perform visualization analysis; genes with cpm (computes counts per million) < 1 or expressing in less than half of the samples were discarded.

### Identifying genes related to tumor stage of LSCC with WGCNA

To explore genes related to the tumor stage of LSCC; we separated LSCC patients into two groups (early: stage I/II and advanced: III/IV); differentially expressed genes (DEGs) analysis between two groups was conducted with the *limma* package using HTSeq counts matrix; to obtain sufficient genes for network construction via WGCNA, we selected gene set using cut-off of *P* value < 0.5. Samples with selected genes expression were clustered to identify outliers; clinical features were integrated with matched samples. In this study, we imparted gender and tumor stage for network construction; the clinical traits were mapped to the clustering tree of samples. The relation of soft power to connectivity and scale-free topology was analyzed and depicted to choose optimal soft power value for constructing the network. Adjacencies were analyzed using the chosen soft power; adjacencies were then transformed into the Topological Overlap Matrix (TOM); the dissimilarity of TOM (dissTOM) was then calculated with TOM; a hierarchical clustering tree was conducted with ‘hclust’ function of WGCNA to depict the clustering of candidate genes; module of individual tree branches was identified with ‘dynamicTreeCut’ function; considering the potential existence of genes with similar expression among different modules identified by ‘dynamicTreeCut’, we evaluated the whole modules co-expression similarity by calculating the correlation between eigengenes and cluster and merged those potential modules with a correlation of 0.75. To gain larger modules, we set the minimum size of modules as 30. The correlation between identified modules and clinical traits was calculated and depicted through heatmap; the relation of Gene Significance (GS) and module membership (MM) was calculated to evaluate the association between significant genes and tumor stage in identified modules.

### Function enrichment analysis of LSCC-related genes

To explore the function of LSCC-related genes, we performed function enrichment analysis; considering the various roles of genes in different modules, we performed enrichment analysis for genes in each module, respectively. A R package clusterProfiler [14] was utilized for enrichment analysis; the name of genes was transformed into ‘ENTREZID’ format for GO enrichment and ‘UNIPROT’ format for KEGG analysis. Top ten terms of each category were visualized using bar plots. We set the minimal size of Ontology term-annotated genes as 5; *p*-value and q value were set to 0.05 for analyzing significantly enriched terms.

### PPI analysis of LSCC-associated genes

Interactions among genes in each module were predicted and visualized using STRING database (https://string-db.org/) and Cytoscape software (https://cytoscape.org/), respectively. Genes with similar expression in the same module might participate in certain roles in response to LSCC by forming gene clusters; we predicted gene clusters based on PPI with ‘MCODE’ function of Cytoscape with default parameters; the first cluster in each module was color-labeled.

### Exploring potentially prognostic biomarkers for LSCC

Univariate Cox regression was performed with survival and depicted with survminer package [15] to analyze the prognostic significance of tumor stage-related genes; genes with *P*-value < 0.05 were considered as potentially significant genes and depicted with forest plot. To evaluate jointly the impact of these significant genes on survival, we conducted multivariate Cox regression analysis with the Cox regression model (according to the manual of survival package) to assess multiple risk factors simultaneously and depicted the result with forest plot; we visualized prognostic significance of candidate genes with Kaplan-Meier (KM) plot.

### Immune infiltration by CIBERSORTx analysis

CIBERSORTx (https://cibersortx.stanford.edu/) is a web-based tool for analyzing the proportion of different types of immune cells. The gene expression matrix of LSCC patients was submitted to CIBERSORTx to predict the fraction of immune cells; the distribution of immune cells among all samples was clustered and depicted via heatmap; we evaluated the potential distribution of immune cells in isolated LSCC patients in the early and advanced stages through PCA plots.

To analyze the impact of genes expression to immune cell distributions, we divided the LSCC patients into two groups based on the median expression of LSCC-related genes with prognostic significance.

### Comparing expression of candidate genes in various tumor stages

To analyze different levels of candidate gene expression, we examined the difference between two generalized tumor stages (the early and advanced stage) with a T-test; the variety of gene expression among relatively exact stages (stage I, stage II, stage III, and stage IV) was tested with Kruskal-Wallis method. We visualized the comparison with ggplot2.

### GSEA analysis of candidate genes

In order to explore function of candidate genes, we calculated median of gene expression and separated LSCC patients into two groups according to the expression of candidate genes (larger or smaller than median expression); GSEA was performed with clusterProfiler package to analyze enriched pathways between two groups with different expression levels of candidate genes; the minimal size of the gene set was set to 50; the cut off of *P-*value was 0.05; top 3 enriched pathways were depicted with ‘gseaplot2’ function.

### Immunohistochemistry

A total of 40 samples were collected from LSCC tissues removed from hospitalized patients at the Sir Run Shaw Hospital affiliated to Zhejiang University Medical College, China, from January 2019 to October 2021. All the patients were informed about the experiments and signed informed consent. The tissue-associated experiments were approved by the Medical Ethics Committee of Sir Run Shaw Hospital affiliated to Zhejiang University Medical College. The 40 samples comprised 12 samples of early LSCC patients and 28 samples of advanced LSCC patients. No patient received chemotherapy or hormone therapy before surgery [16].

The expression of COPB2 and RYK in the tissue samples was explored by using immunohistochemical staining (IHC). IHC staining was performed according to the manufacturer’s instructions [17]. Tissue samples were fixed in 10% formalin embedded in paraffin and cut into slices (4μm). The primary antibodies were obtained from ThermoFisher Scientific: COPB2 (#PA5-96557) and RYK (#PA1-30319). The sections were visualized with InvitrogenTM EVOS FL Auto 2 Fluorescence Microscopic Imaging System. The prop.test of “stats” package in R language (version:4.1.3) is used for statistical analysis of positive expression rate (%) and high-expression rate (%).

## Results

### Networks construction and modules identification

**Fig. 1:**
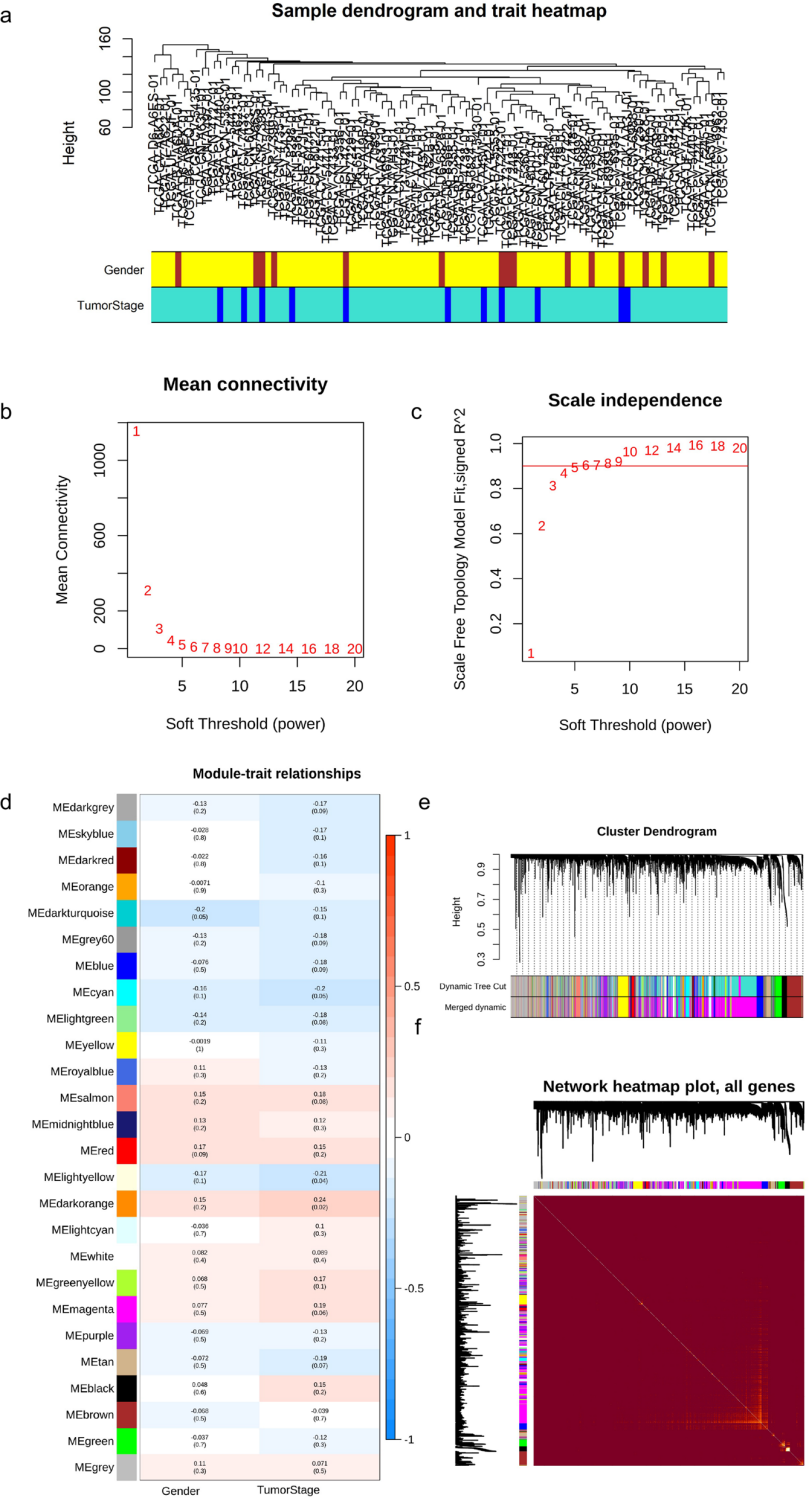
Networks construction using weighted gene co-expression network analysis (WGCNA). **a** the clustering tree of samples and matched clinical traits; brown blocks in ‘Gender’ refer to female, yellow to male samples; bule blocks in ‘TumorStage’ indicate LSCC patients in early stage, while turquoise represents advanced stage. **b** and **c** the soft threshold value was determined based on mean connectivity and scale-free fit. **d** The tumor stage of LSCC and modules relationships were depicted; digits in the boxes were the correlations (up) and corresponding *P*-value (down); **e** The cluster tree and identified modules were pictured. **f** The network heatmap plot of all genes

**Fig. 2:**
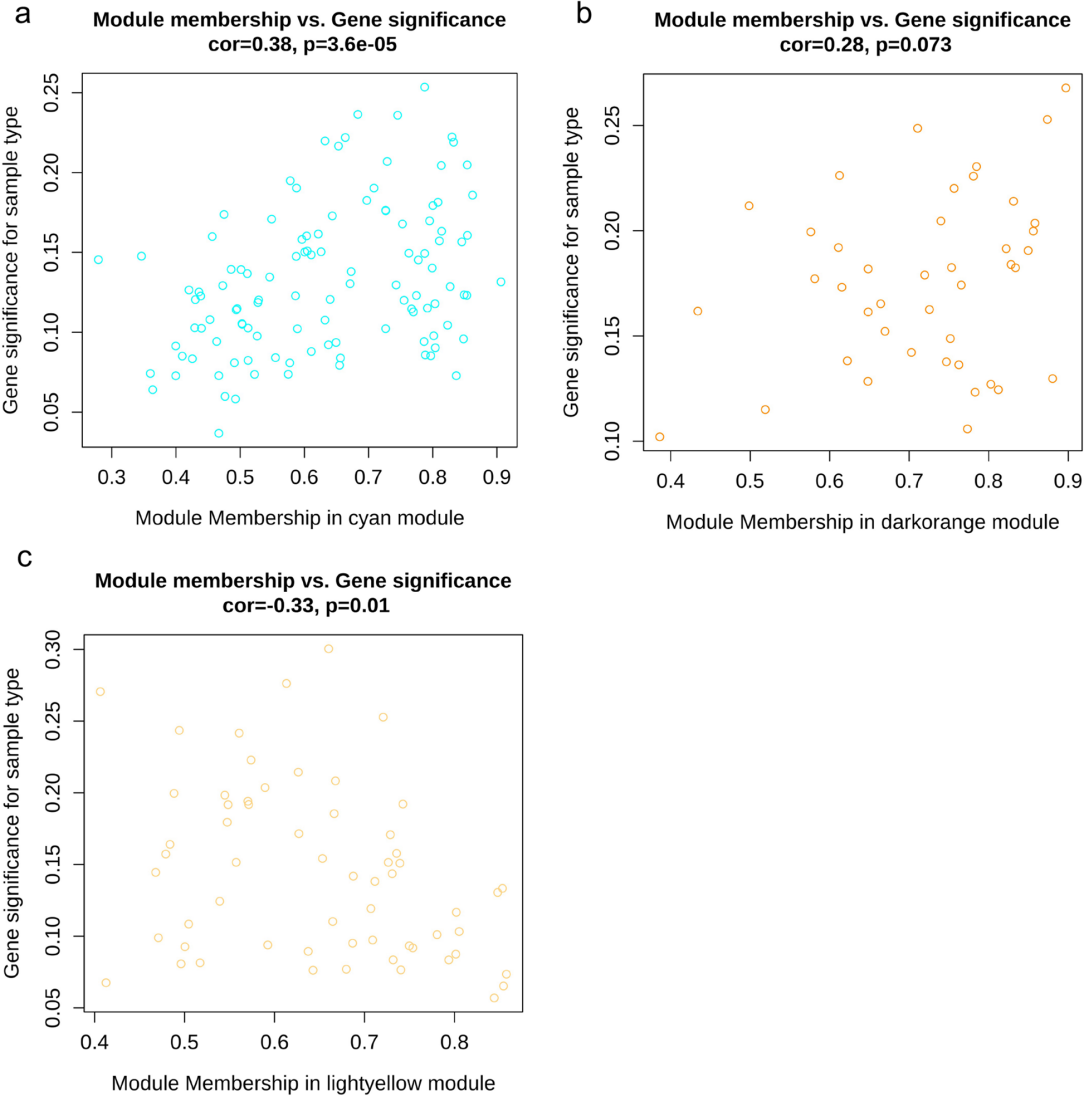
Scatterplots of module membership (MM) *vs.* gene significance (GS) related to LSCC stages. **a**, **b**, and **c** referred to the scatterplot of GS *vs.* MM in the module cyan, darkorange, and lightyellow, respectively. The X-axis indicated the correlation of module eigengene and the gene expression; the Y-axis showed the correlation between gene expression profile and traits (tumor stages); each dot represented a gene

We obtained a matrix formed with 96 LSCC patients (85 in advanced and 11 in early tumor stage) and 60483 genes by collecting and integrating expression matrix associated with LSCC; clinical summary of LSCC patients was descripted in Table 1. A genes set containing 6891 genes (including 6278 mRNAs and 322 lncRNAs) was collected with *P*-value < 0.5 for network construction. The dendrogram of clustering samples indicated no obvious outliers. The correlation of samples and clinical traits was depicted in the clustering dendrogram; no significant separation of clustering samples was observed from the image according to gender or tumor stage (Fig. 1a). We observed a dramatic decrease along with the increase of soft power before β = 9 (Fig. 1b) and we considered β = 9 as the suitable soft power value for constructing the network, since it is the lowest values where the scare-free topology fit reached 0.9 (Fig. 1c). A total of 25 modules were obtained from the network constructed by WGCNA, with gene counts ranging from 34 to 1955 (Fig. 1c, e). Three modules—module ‘cyan’ (cor = -0.2, *P* = 0.05), module ‘darkorange’ (cor = 0.24, *P* = 0.02), and module ‘lightyellow’ (cor = -0.21, *P* = 0.04)—were related to tumor stage of LSCC (Fig. 1d). Topological Overlap Matrix (TOM) among collected genes in this study was depicted with heatmap; higher overlap was marked with bright yellow color (Fig. 1f). 112 genes in module ‘cyan’, 42 genes in ‘darkorange’, and 60 genes in ‘lightyellow’ were collected for further analysis. A statistically significant correlation between GS and MM was observed in two modules (module ‘cyan’ with cor = 0.38 and *P* = 3.6e-05; module ‘lightyellow’ with cor = -0.33 and *P* = 0.01) (Fig. 2); genes in module ‘cyan’ with high significant correlation with tumor stage also played crucial roles (Figs. 1d and 2a).

**Table 1:** Summary table of clinical traits of LSCC patients

		**Advanced stage**	**Early stage**	***P* value**	**SMD**
N		85	11		
Gender (%)	female	11 (12.9)	4 (36.4)	0.066	0.565
	male	74 (87.1)	7 (63.6)		
Age (days) at diagnosis (median [IQR])		22577.00 [20433.00, 24702.00]	22796.00 [21790.50, 27982.00]	0.216	0.367
Vital status (%)	alive	51 (60.0)	6 (54.5)	0.753	0.110
	dead	34 (40.0)	5 (45.5)		

*SMD* Standardized mean differences; considering the nonnormal distribution of age at diagnosis, its significance (*P* value) to tumor stage was calculated with Kruskal-Wallis rank sum test; the significance of other two traits (gender and vital status) and tumor stage was performed with Fisher’s exact test

### Functional enrichment analysis of LSCC-related modules

**Fig. 3:**
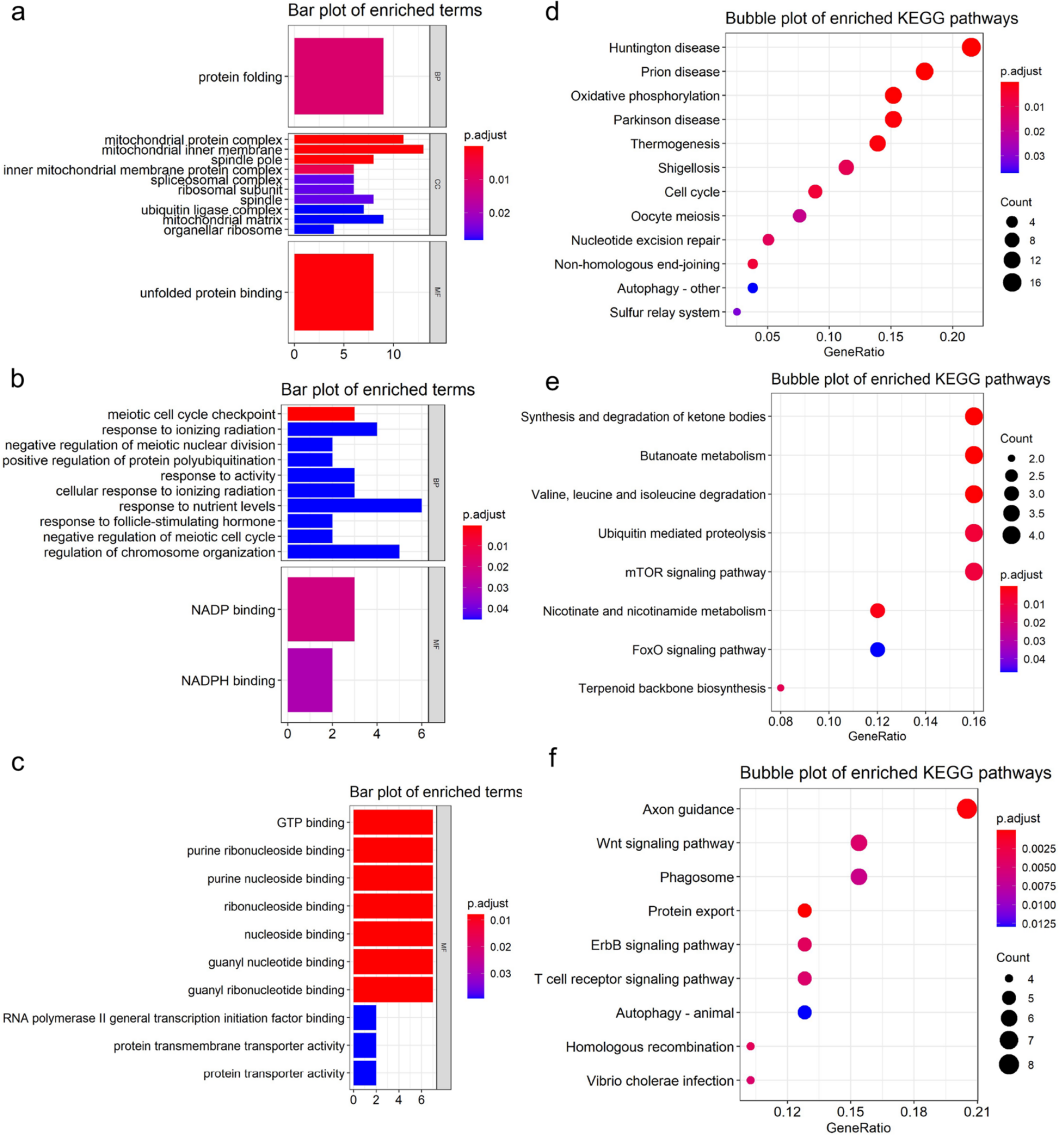
Functional enrichment analysis of genes in three significant modules. **a**, **b**, and **c** represented the bar plot of Gene Ontology terms of cyan, darkorange, and lightyellow modules, respectively. Top ten terms of each category were visualized. BP for biological process, CC for cellular component, and MF for molecular function. **d**, **e**, and **f** showed the KEGG enrichment of genes in module cyan, darkorange, and lightyellow, respectively. The dot size refers to the count of genes in each pathway. The color bar indicated the significant level

Genes in module ‘cyan’ were enriched in 27 GO terms (1 in BP, 25 in CC, and 1 in MF), mainly focused on the cellular components such as mitochondrial inner membrane, mitochondrial protein complex (Fig. 3a). Module ‘darkorange’ was mainly enriched in biological process terms (12 terms in BP and 2 terms in MF) such as response to ionizing radiation and nutrient levels (Fig. 3b); Genes in module ‘lightyellow’ were fully enriched in 11 MF terms (Fig. 3c), especially in binding function like GTP binding and ribonucleotide binding. KEGG enrichment analysis showed that modules ‘cyan’, were significantly enriched in 12 pathways such as oxidative phosphorylation and cell cycle (Fig. 3d), 8 of module ‘darkorange’ such as ubiquitin mediated proteolysis and mTOR signaling (Fig. 3e), and 9 of ‘lightyellow’ such as Wnt signaling pathway (Fig. 3f).

### PPI of LSCC-related modules

**Fig. 4:**
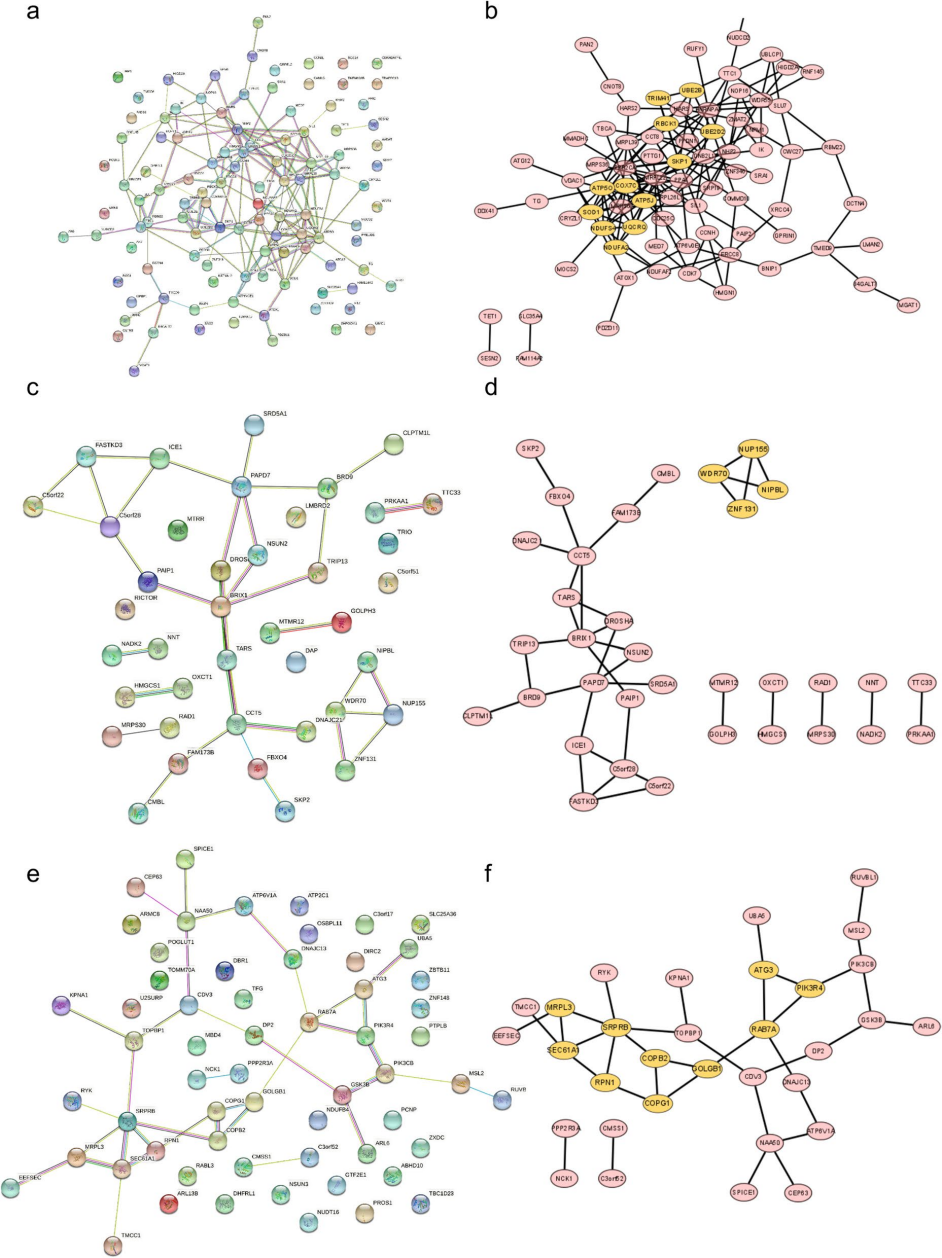
Protein-protein interaction (PPI) of genes related to LSCC tumor stage. **a** and **b** referred to the PPI of genes in the cyan module and predicted clusters (top 3 were showed), **c** and **d** to the darkorange module, and **e** and **f** to the lightyellow module. The first predicted genes cluster were color-coded in yellow

Gene clusters of LSCC-related genes with similar expression patterns were predicted using MCODE function of Cytoscape; predicted gene clusters with the highest score were color-labeled (Fig. 4). Genes associated with LSCC were observed in the cluster such as *SOD1* and *SKP1* in module ‘cyan’ (Fig. 4a, b), *NUP155* and *NIPBL* in module ‘darkorange’ (Fig. 4c, d), and *SEC61B*, *SEC61A1*, and *PIK3R4* in module ‘lightyellow’ (Fig. 4e, f).

### Overall survival analysis of genes in LSCC-related modules

**Fig. 5:**
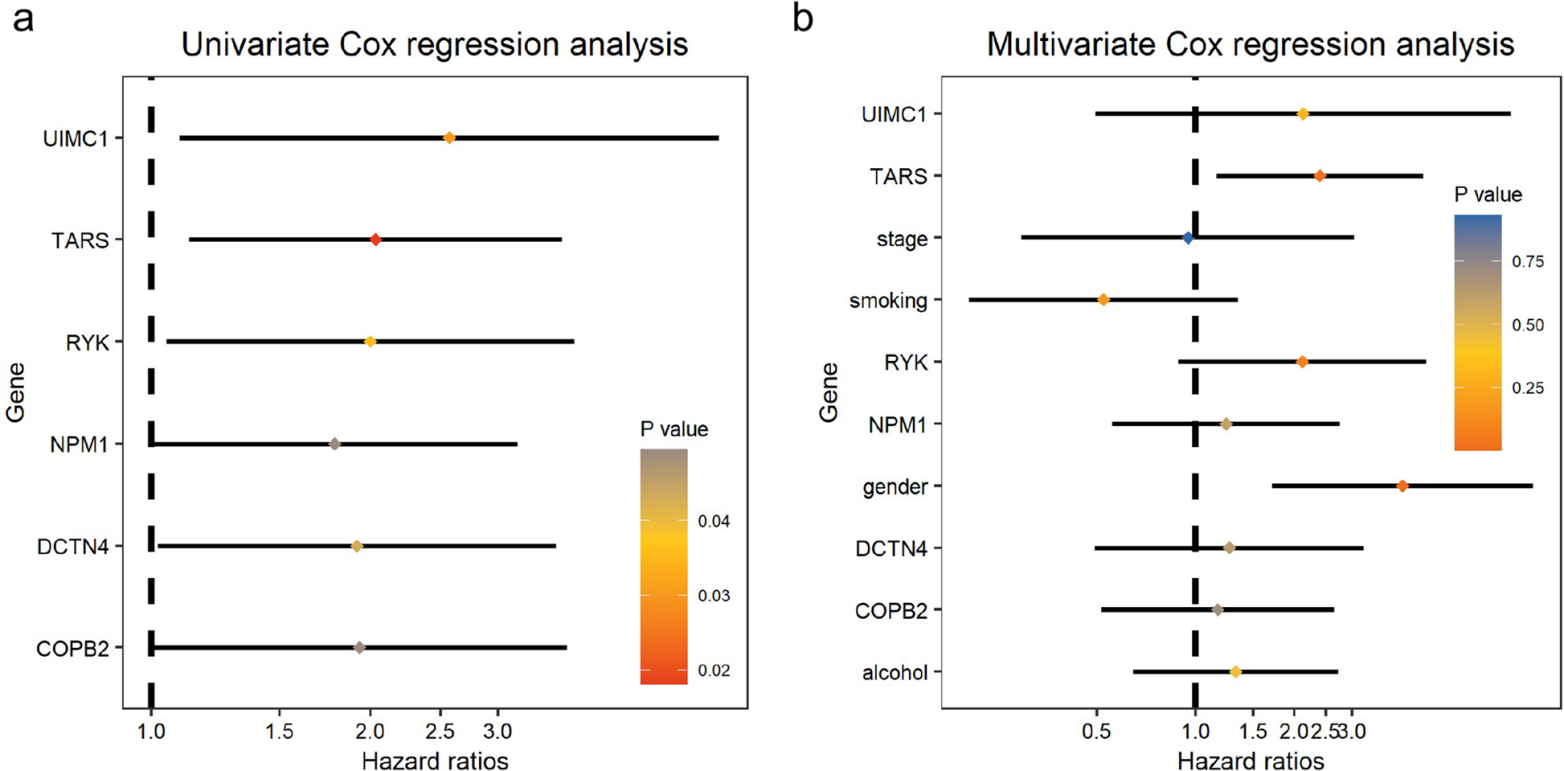
Cox regression analysis to explore potentially prognostic biomarkers for LSCC. Univariate (**a**) and multivariate (**b**) Cox regression analysis of genes from three significant modules

Three genes (*UIMC1*, *NPM1*, and *DCTN4*) in module ‘cyan’, one gene (*TARS*) in module ‘darkorange’, and two genes (*COPB2* and *RYK*) in module ‘lightyellow’ showed statistically significant relation to overall survival with *P*-value < 0.05 with univariate Cox regression (Fig. 5a). Hazard rates (HR) of genes were larger than one which indicated a negative correlation between gene expressions and survival rates: higher gene expression level with lower survival probability. Multivariate Cox regression analysis showed the significance of the overall model, of which significant values of three methods (Likelihood ratio test, Wald test, and Logrank test) ≤ 0.02; only one gene (*TARS*) showed independent prognostic significance with HR = 2.40 and *P* = 0.02 (Fig. 5b).

### Alternation of immune cells between different stages

**Fig. 6:**
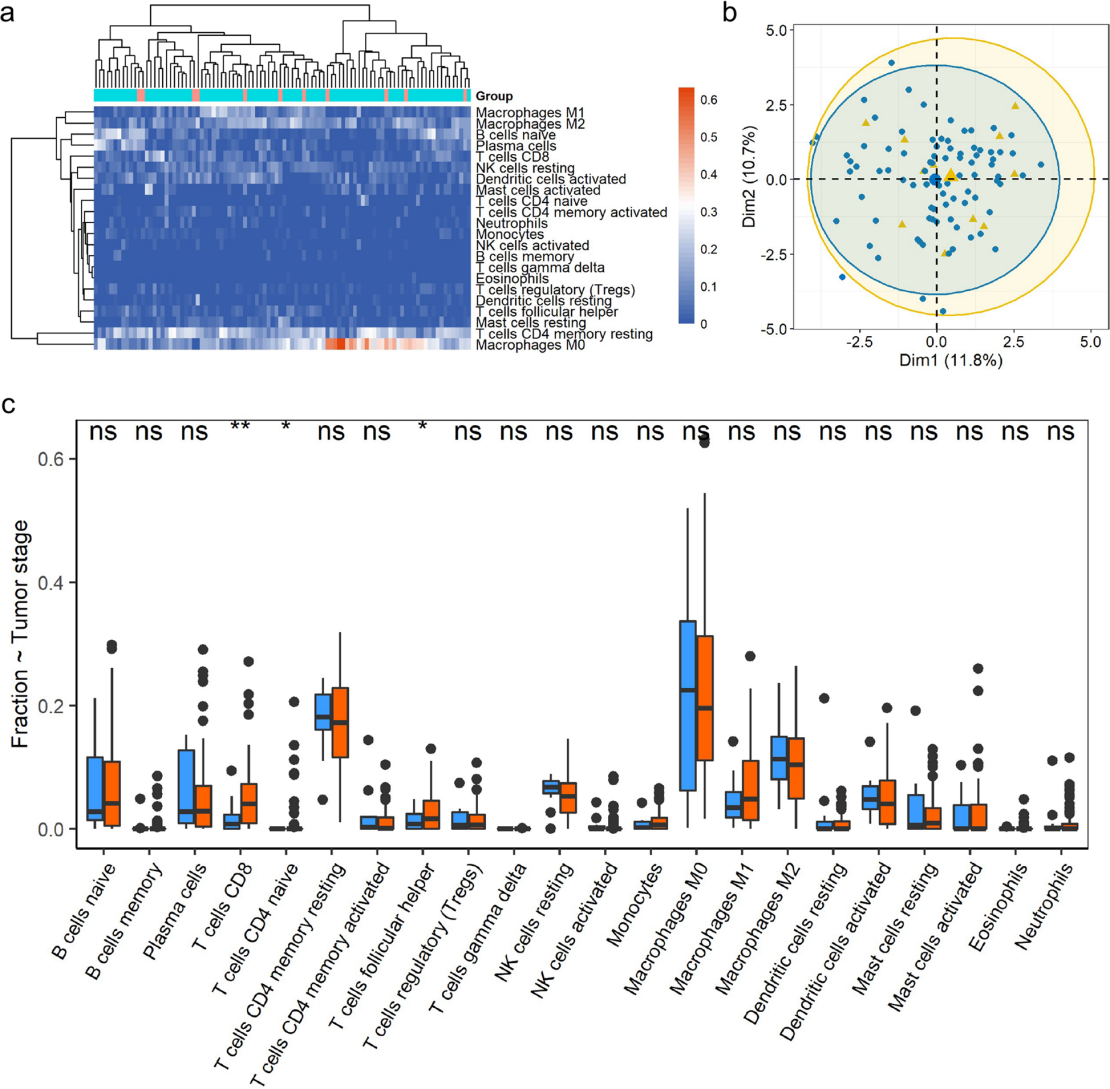
Immune analysis of different tumor stages. **a** The proportion of immune cells in two tumor stages (red refers to early and turquoise to advanced stage) was visualized with heatmap. **b** The power of distribution of immune cells in separating different tumor stages was depicted with principal components analysis plot. **c** Fraction of immune cells in different stages was showed with boxplots; blue refers to early stage and red to advanced stage

Two stages of LSCC patients were not obviously separated as shown in the heatmap and PCA plot (Fig. 6a, b); the fractions of three immune cells showed variety between two groups: the distribution of CD8+ T cells was significantly increased in advanced stages; CD4+ T cells (naive) was accumulated in LSCC patients in advanced stages; the proportion of T cells follicular helper was increased in advanced LSCC patients (Fig. 6c).

### LSCC-related genes engaged in the immune response

**Fig. 7:**
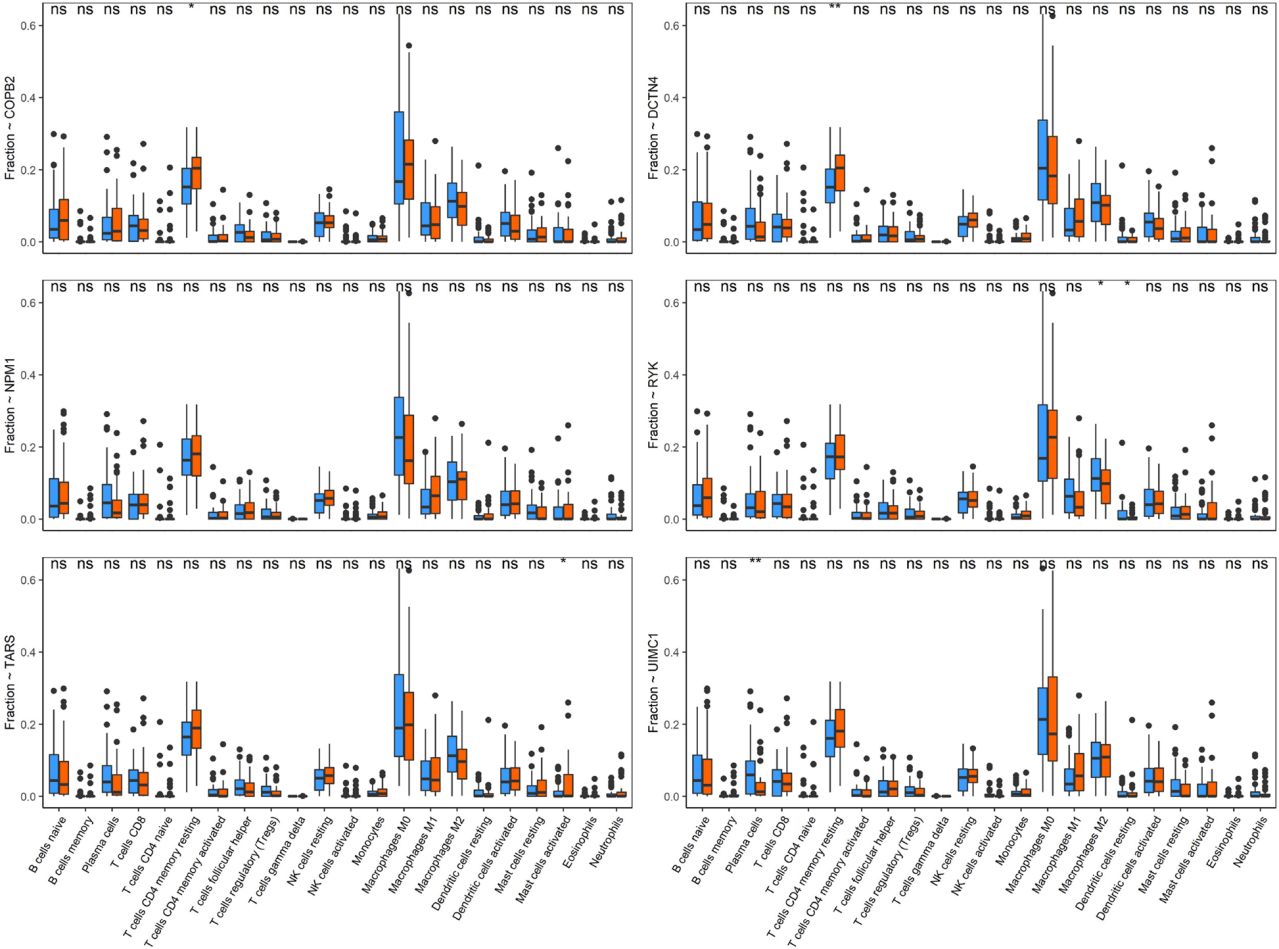
Distribution of 22 differentially infiltrated immune cell. The boxplots indicate the different proportion of 22 immune cells in two patient groups divided with median expression of 6 genes. Red refers to LSCC patients with relatively higher expression, blue to patients with lower expression. The significant levels at 0.01, 0.05, and 1 were symbolized with “**”, “*”, and “ns”, respectively

Compared with lower (blue boxes) expression of five genes (*COPB2*, *DCTN4*, *RYK*, *TARS1*, and *UIMC1*), LSCC patients with higher expression (red boxes) represented significantly different fraction of immune cells (Fig. 7). Compared with patients with lower *COPB2* and *DCTN4* expression, the fraction of T cells CD4 (memory resting) was statistically increased in LSCC patients with higher expression; increasing expression of *RYK* showed association with downregulation of macrophages (M2) and Dendritic cells resting; the *TARS1* expression presented association with the upregulation of fraction of mast cells (activated); the proportion of plasma cells was statistically decreased in LSCC patients with higher expression.

### Various expression patterns of candidate genes among different tumor stages

**Fig. 8:**
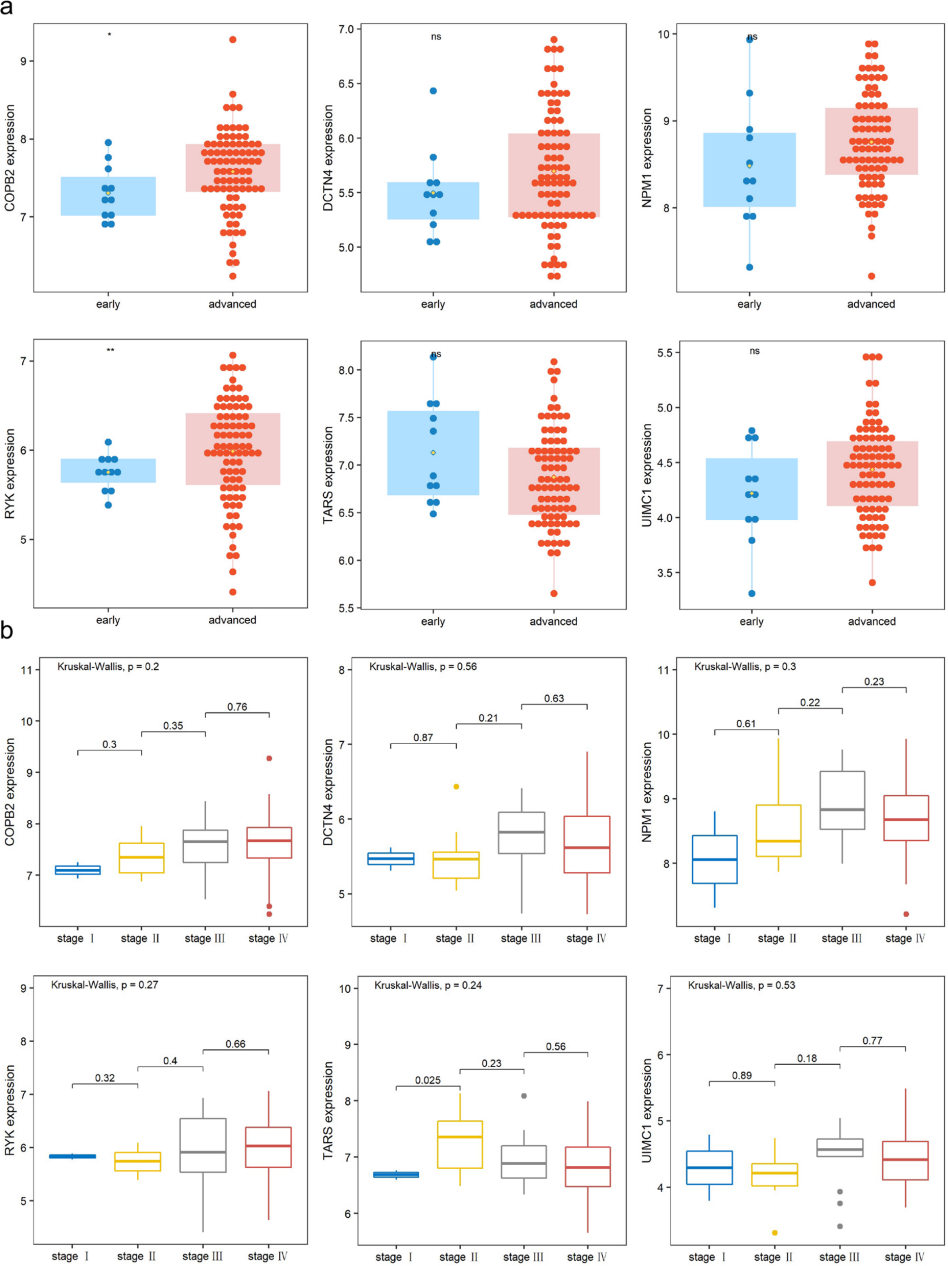
Comparing crucial genes expression in different tumor stages. **a** The comparison of critical genes between early and advanced stages was depicted with boxplots. **b** Gene expression among four stages (from stage I to stage IV) were visualized with boxplots

We observed that two genes—*COPB2* and *RYK*—showed statistically significant expression change between two groups; the expression of *COPB2* and *RYK* significantly increased in patients with advanced LSCC compared to those with early stages (Fig. 8a). The expression of *TARS* and other genes showed difference (not significantly) between two stages (Fig. 8a). We noticed the constant increasing of *COPB2*, though not significant between adjacent stages, in the first three status of LSCC (Fig. 8b); despite the difference among early and advanced stages, the expression of *RYK* showed no obvious change among different tumor stages of LSCC patients; *TARS* expression was significantly stimulated in the stage II compared with stage I (Fig. 8b).

### Enriched pathways related to *COPB2 *and *RYK*

**Fig. 9:**
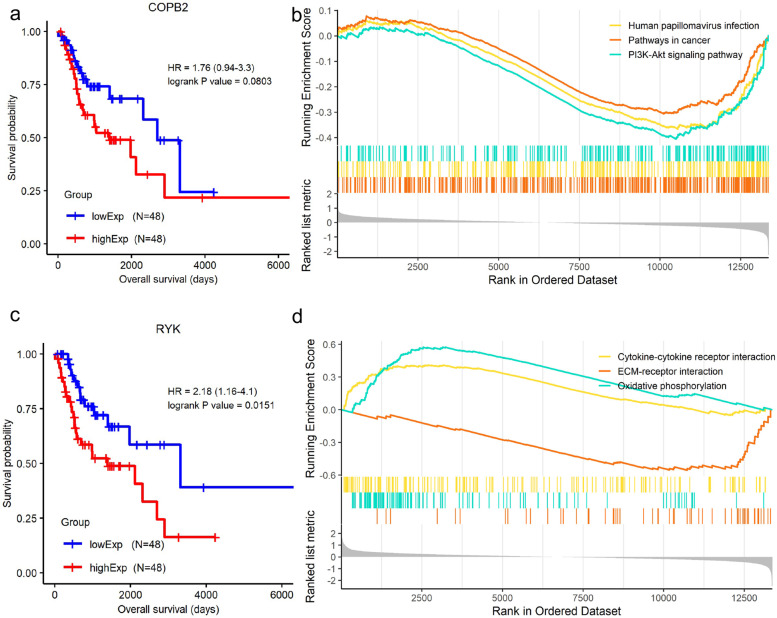
Prognostic significance and gene set enrichment analysis (GSEA) of candidate genes. **a** and **c** refer to the KM plots of COPB2 and RYK, respectively; **b** and **d** represent GSEA of COPB2 and RYK, respectively

We examined and visualized the prognostic significance of *COPB2* and *RYK* with KM plot and found that *COPB2* showed no statistically prognostic value under KM with ‘median’ separation strategy (Fig. 9a). *RYK* showed a significant correlation with the prognosis of LSCC patients under the KM method (Fig. 9c). Multiple pathways associated with cancers such as pathways in cancer, PI3K-Akt signaling pathway, and Cytokine-cytokine receptor interaction, were observed to be enriched among LSCC patients with various expression of *COPB2* and *RYK* (Fig. 9b, d).

### Validation of *COPB2* and *RYK*

**Fig. 10:**
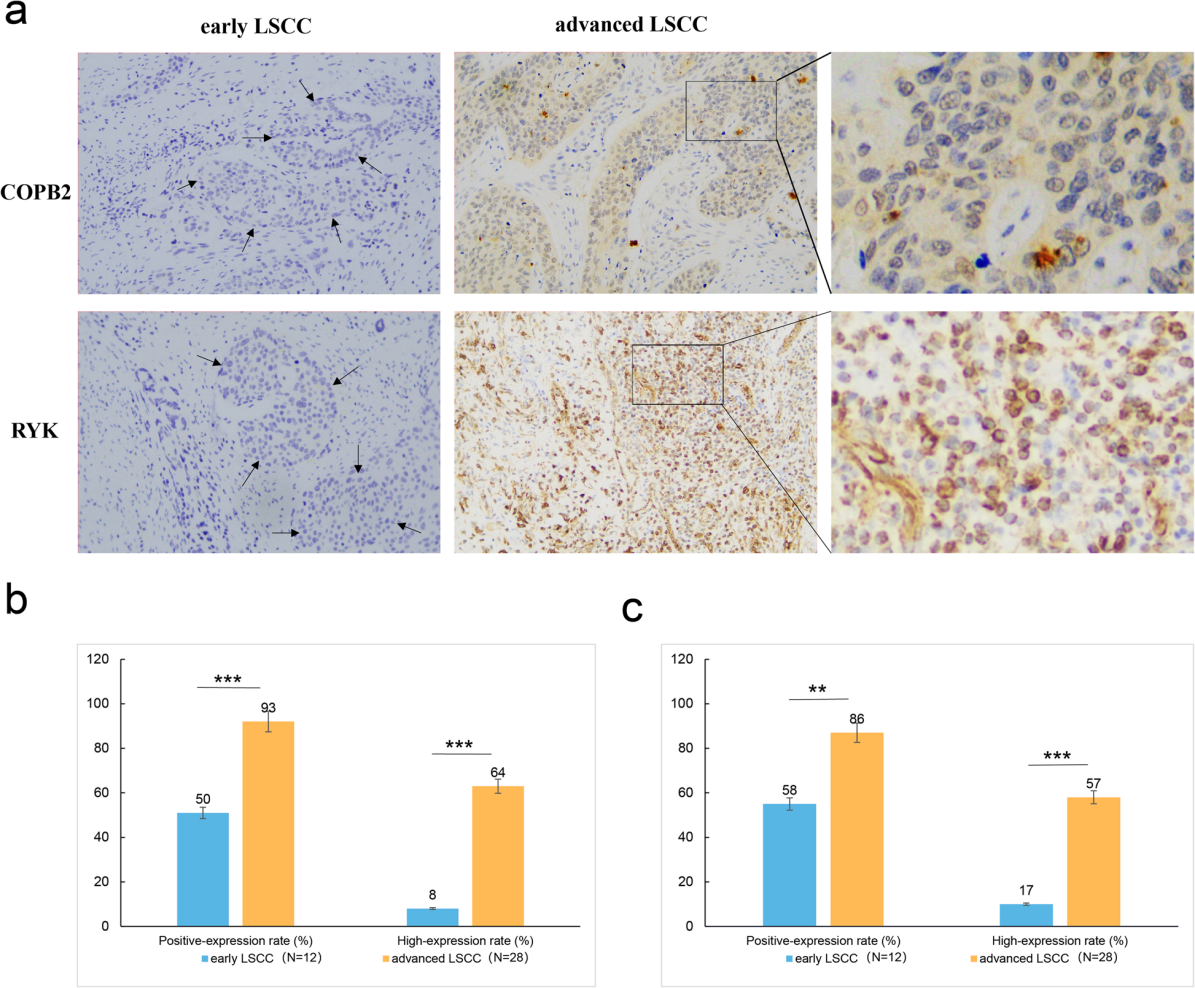
The expression of COPB2 and RYK in LSCC patients. **a** represent the results of immunohistochemistry analysis (200×, 400×), and arrows point to tumor cells; **b** represent positive-expression rate and high-expression rate of COPB2; **c** represent positive-expression rate and high-expression rate of RYK. Compared with early LSCC, ^**^P<0.01, ^***^P<0.001

Based on the above analysis, the expression of *COPB2* and *RYK* significantly increased in patients with advanced LSCC compared to those with early stages. The results of immunohistochemistry analysis showed that COPB2 and RYK appeared brownish-yellow in tumor tissues of advanced LSCC patients (Fig. 10a). What’s more, COPB2 was expressed in cytoplasm; RYK was mainly expressed in cytomembrane. COPB2 had an expression rate of 92% and a high-expression rate of 63%, which were significantly higher than in early LSCC patients (51% and 8%) (All *P* <0.001) (Fig. 10b). RYK had an expression rate of 87% and a high-expression rate of 58%, which were significantly higher than in early LSCC patients (55% and 10%) (*P* = 0.002, *P* <0.001) (Fig. 10c). In a word, the results of COPB2 and RYK were consistent with those of bioinformatics analysis.

## Discussion

Despite the development of therapeutic implement for LSCC such as total laryngectomy or radiotherapy, LSCC, especially advanced LSCC, has a poor prognosis, and therefore there is a need to identify the modular signatures of different stages of LSCC. In this study, we explored genes associated with LSCC tumor stages with WGCNA using expression matrix of TCGA; 6891 genes obtained with threshold value of *P* value < 0.5 were clustered into 25 modules (Fig. 1); 3 modules were significantly associated with LSCC (Fig. 1). We observed a gene cluster, containing three genes (*UQCRQ*, *NDUFA2*, and *NDUFS4*) with different expression in various tumor stages of larynx squamous cell carcinoma, in modules related to tumor stage (Fig. 4a) [18]. A BTB-ZF protein, ZNF131, is required for early B cell development [19].

Pathways and function process widely reported in LSCC such oxidative phosphorylation, mitochondrial pathway, response to ionizing radiation, and ubiquitin mediated proteolysis were observed in function enrichment analysis (Fig. 3). The mitochondrial pathways were widely reported involved in the apoptosis of LSCC cells mediated by multiple elements such as cetuximab enhanced oridonin [20]. In our study, we noticed the enriched terms of genes in module ‘cyan’ in the cellular components such as mitochondrial inner membrane, and mitochondrial protein complex (Fig. 3a), which indicates genes in module ‘cyan’ might play a role in different stages of LSCC via influencing the apoptosis status of LSCC cells through mitochondrial pathways. The previous publications showed that mTOR and Wnt signaling pathways played an active role in LSCC. The AKT/mTOR was activated by FADS1 and related to the progression of LSCC [21]. Wnt/beta-catenin signaling was regulated by long non-coding RNA, which affects the proliferation of LSCC [22, 23]. Overexpression of Wnt3a, one canonical Wnt/β-catenin signaling pathway, was association with the poor overall survival of LSCC patients [6]. In this analysis, we observed the significant enrichment of mTOR signaling pathway and Wnt signaling pathway of genes in two LSCC stage related modules: ‘darkorange’ and ‘lightyellow’ (Fig. 3d, f); these results indicated the significant role of mTOR and Wnt signaling pathway in the progression of LSCC. Processes associated with autophagy were enriched in genes in module ‘cyan’ and ‘lightyellow’ (Fig. 3b, f). Autophagy suppression was reported to enhance DNA damage and cell death upon treatment with PARP inhibitor Niraparib in LSCC [24]. Deprivation of asparagine triggered cytoprotective autophagy in LSCC [21]. A recent publication reported the inhibition of autophagy in LSCC cells and found that circPARD3, autophagy-suppressive circRNA, promotes the malignant progression of LSCC cells via inhibiting autophagy [25].

*COPB2* was reported to promote cell proliferation and tumorigenesis through up-regulating *YAP1* expression in lung adenocarcinoma cells [26]. *COPB2* gene silencing inhibits colorectal cancer cell proliferation and induces apoptosis via the JNK/c-Jun signaling pathway [27]. In this study, *COPB2* showed a statistically significant relation to overall survival in LSCC via univariate Cox regression (Fig. 5a). The fraction of T cells CD4 (memory resting) was statistically increased in LSCC patients with higher (larger than median expression) *COPB2* expression (Fig. 7). *COPB2* was reported to be closely linked to inflammatory and immune responses and higher immune cell infiltration, which is in line with our result [28]. The expression level of *COPB2* was significantly increased in advanced stages of LSCC compared with that in early stages (Fig. 8a), indicating that *COPB2* may be related to the development of LSCC; we observed rising tendency (though not statistically significant) of *COPB2* expression from stage I to stage III. GSEA showed that expression of *COPB2* was associated with genes involved in the PI3K/Akt signaling pathway (Fig. 9b); further exploration needs to be done to explore the underlying molecular mechanism of *COPB2* in regulating procession of LSCC. Finally, COPB2 appeared high-expression in tumor tissues of advanced LSCC patients based on immunohistochemistry staining (Fig. 10a).

RYK, a receptor of noncanonical Wnt ligand Wnt5a, was actively involved in WNT signaling. WNT/RYK signaling restricts goblet cell differentiation during lung development and repair [29]. RYK was also positively correlated with gastric cancer tumorigenesis and the potential of liver metastasis [30]. In this study, we found that *RYK* expression was statistically significantly enhanced in advanced LSCC patients (Fig. 8a). *RYK* had a statistically significant relation to overall survival in univariate Cox regression analysis (Fig. 5a); the probability of survival was higher in LSCC patients with lower *RYK* expression in KM analysis (Fig. 9c); these indicates the prognostic significance of RYK as biomarker. Wnt5a is a chemokine secreted by inflammatory-activated human macrophages that maintain their inflammatory response in an autocrine manner [31]. As a receptor of Wnt5a, high expression of *RYK* suppressed the accumulation of macrophages (M2) and Dendritic cells resting (Fig. 7), which is consistent with the previous research. GSEA result demonstrated that expression RYK showed correlation with genes involved with cytokine-cytokine interaction, ECM-receptor interaction, and oxidative phosphorylation (Fig. 9d). ECM-receptor interaction was considered as potential role in participating in multiple tumor biology such as motility, invasion, and metastasis process of LSCC [32]. Researchers found that silencing of the WNT-5A receptors Frizzled 8 (FZD8) and RYK attenuated TGF-β-induced ECM expression [33]; future work needs to be done to analyze the expression status of RYK in different tumor stages and its role in progression of LSCC. Finally, RYK appeared high-expression in tumor tissues of advanced LSCC patients based on immunohistochemistry staining (Fig. 10a).

In conclusion, two genes, *COPB2* and *RYK* were found to be significantly correlated with tumor stages of LSCC and represented negative correlation with overall survival of LSCC patients.

## Data Availability

The datasets generated and/or analysed during the current study are available in the TCGA database, [https://portal.gdc.cancer.gov/projects/TCGA-HNSC].
